# Supplementary material for: Are carbon emissions trading and green financial instruments synergistic? -Comprehensive quantitative research based on content analysis
Source: PLoS One. 2024 Mar 7;19(3):e0298601. doi: 10.1371/journal.pone.0298601 (PMC10919601; doi:10.1371/journal.pone.0298601)
Supplement: S1 Data — (ZIP) [file pone.0298601.s001.zip › Data/地级市Z_F工作报告.zip!/╣≤╓▌/╣≤╤⌠╩╨/└┤╫╘╣1⁄2╓┌║┼í╛┬φ┐╦╩2╛▌═°í┐_2010.docx]

各位代表：

我代表市人民政府，向大会作工作报告，请予审议，并请各位政协委员和其他列席人员提出意见。

2009年政府工作回顾

过去的一年，是我市经济社会发展极为困难的一年。面对国际金融危机的严重冲击，我们在省委、省政府和市委的正确领导下，在市人大、市政协的监督、支持和帮助下，高举中国特色社会主义伟大旗帜，以邓小平理论和“三个代表”重要思想为指导，深入贯彻落实科学发展观，以加快生态文明城市建设为总抓手，按照“应挑战、保增长、重民生、推改革、促开放、善领导”的要求，解放思想、埋头实干，迎难而上、共度时艰，全市经济又好又快发展，人民生活继续改善，社会保持稳定，全面完成了市十二届人民代表大会第四次会议确定的各项目标任务。

——全力保增长，经济又好又快发展。全市生产总值902.61亿元，比上年增长13.3%，其中：一产增加值50.07亿元，增长8.1%；二产增加值402.24亿元，增长12.6%；三产增加值450.3亿元，增长14.5%。财政总收入252.05亿元，增长12.4%，剔除政策性减收和不可比因素后，增长19.1%；地方财政收入105.36亿元，增长18.3%，剔除政策性减收和不可比因素后，增长23%。地方财政支出168.98亿元，增长18.8%。居民消费价格指数为97.7，下降2.3%。单位生产总值能耗下降4%左右；化学需氧量排放控制在5.07万吨；二氧化硫排放量控制在17.9万吨。

——倾力保民生，人民生活进一步改善。城市居民人均可支配收入15041元，增长8.9%；农民人均纯收入5316元，增长10.3%。新增就业岗位5.2万个，城乡统筹就业7.8万人，城镇登记失业率3.5%，96.6%的返乡农民工、5.9万名高校毕业生实现就业。

——奋力保稳定，平安贵阳建设成效明显。信访维稳、安全生产和应急管理工作全面加强，连续4次、16年荣获全国社会治安综合治理优秀地市荣誉称号，市民安全感比上年提高4个百分点以上,建国60周年等重大节庆活动中社会稳定。安全生产事故起数和死亡人数分别为784起、344人，分别下降10.4%、10.4%，实现“双下降”。

一年来，我们始终以科学发展观为统领，重点抓了七个方面的工作：

（一）投资带动保增长，重大项目建设有新突破

固定资产投资完成782.64亿元，增长30.1%，投资对经济增长的贡献率达52%以上。完成“投转固”项目48个，涉及资金18亿元。在国家下达扩大内需项目中，共获得29.05亿元中央投资支持，市属283个项目已开工272个，完工179个，开工率、完工率分别达96.1%、63.3%，带动各级各类配套资金70余亿元。城市发展基础极大夯实。市域快速铁路已开工白云至龙里北联络线等5个项目；城市轻轨一号线已开工建设市政配套工程会展中心车站；全长121公里的环城高速公路全线通车。甲秀南路、北京西路、机场路、水东路、黔灵山路等10余条城市主干道建成。启动息烽港区水运码头建设。集中实施了一批龙头型、基地型产业项目。兴建首钢贵阳特殊钢新特材料循环经济工业基地、中航工业贵阳产业基地、贵州广铝年产80万吨氧化铝、贵航军转民高新技术产业园等一批重大工业项目。贵阳国际会展中心、奥体中心主体育场等项目建设进度加快。实施3000万元以上产业化项目74个，其中亿元以上项目41个。城市保障能力得到加强。华电清镇塘寨电厂2×60万千瓦机组项目顺利推进，赵斯变电站建成供电，完成电网建设投资21.4亿元；日供气30万立方米的华能焦化四号焦炉、日处理污水25万吨的新庄污水处理厂建成投入使用；开工建设鱼洞峡水库；贵阳护理职业学院建成；贵阳市数字化城市综合管理系统投入使用。

（二）创新引领促转型，产业整体竞争力有新提升

加快发展服务业。社会消费品零售总额达412.72亿元，增长20.1%，服务业占全市生产总值的比重达49.9%。旅游业持续高速增长。赴北京、台北、上海、重庆、广州等地开展旅游推介，相继掀起“百万游客游贵阳”、“万名台湾游客游贵阳”及省内“百元悠游贵阳”热潮。开通贵阳至台北定期航班、贵阳至普吉岛包机等新航线，旅游专列、包机大幅增加，龙洞堡机场运送旅客数突破580万人。接待海内外游客3288.47万人次，旅游总收入294.85亿元，分别增长25.2%、57.4%。金融业发展实现重大突破。中信银行贵阳分行开业，招商银行等5家银行获批在我市设立分行，开设一批政策性担保公司、村镇银行和小额贷款公司。物流节点城市建设开局良好。抢抓我市列为国家区域性物流节点城市的机遇，建成扎佐物流园区铁路战略装车点及宝通工业物流园主体工程等一批物流项目，西南物流中心进入中国物流园区50强，获得全国物流中心城市杰出成就奖。城乡市场体系建设迈上新台阶。红星美凯龙家居广场、恒峰步行街建成开业，新建、改造一批农贸市场、社区连锁超市及“万村千乡市场工程”项目，新增3个全国社区商业示范社区和21个商务部标准化菜市场示范项目。房地产业快速健康发展。房地产开发投资210.33亿元，增长23.6%；商品房销售面积818.16万平方米，增长98.6%；销售额307.49亿元，增长137%。

打好工业提速增效攻坚战。认真落实振兴工业经济大会精神，强力推进工业强市战略，工业经济逆势而上，从9月起大幅回升，单月增幅均达15%以上。全年工业增加值达304.49亿元，增长10.3%。园区建设进展加快。编制完成九大工业园区控制性详细规划。高新区区位调整获得国务院批准，顺利推进沙文生态工业园金苏大道、麦沙大道建设，基本完成贵航军转民高新技术产业园“八通一平”。加快园区项目建设，开磷集团120万吨磷酸二铵工程一期60万吨、青利天盟7万吨尾气生产甲酸钠、息烽2×5亿块磷石膏标砖等项目顺利投产。培育大产业、大企业成效明显。突出抓好“四个”十大重点企业和重点工业项目，开磷集团、中铝贵州分公司等重新满负荷生产，贵阳卷烟厂、贵州轮胎厂和三大军工企业增长较快。通过高新技术企业重新认定53家，高技术工业增加值增长20.4%。十大重点制药企业工业增加值增速均超过30%，制药业产值突破100亿元大关，增长26.3%。一批重点项目达产增效，振华公司年产1000吨锂离子电池正极材料生产线一期工程、安大宇航新材料环形锻件生产线、老干妈春梅酿造公司二期技改等重点项目建成投产。建成1000万元以上工业项目65个，达产后新增产值逾110亿元。“小巨人企业成长计划”扎实推进。规模以上工业企业由520家增加到550家。

抓好创新能力建设。实施科技创新行动计划，市本级财政科技投入1.4亿元，规模以上企业研发投入6.13亿元。中科院地化所矿床地球化学实验室、航天林泉公司精密微特电机技术实验室、复合改性聚合物材料工程技术中心、贵阳软件产业基地、科创新材料生产力促进中心跻身“国家级”行列，三占公司新型优质凿岩钎具产业化等26个项目获得国家科技专项资金支持。重点支持贵阳数字产业园发展，动漫软件产业增加值增长50%。第五次荣获国家科技进步考核先进市。强力推进知识产权创造和运用、管理服务、保护和宣传培训“四大体系”建设，4个工业园区、100家企业开展知识产权工作试点，专利申请量、授权量分别达2465件、1336件，分别增长28.9%、27%，荣获国家知识产权工作示范城市称号。

（三）规划引领强品质，城市美化绿化亮化净化有新进展

规划龙头作用进一步增强。编制完成《贵阳市城市总体规划（2009—2020年）》、《贵阳市土地利用总体规划（2006—2020年）》，完成20余项控规及城市设计。严格规划执法检查，治理乱搭乱建和乱设户外广告初见成效，实现临时建筑“零审批”。

新一轮城市建设全面展开。重大基础设施投资规模大、项目多、施工面广，西南环线、金朱东路、兴筑东路等城市道路建成通车，拓宽改造朝阳洞路二期、黔江北路、五里冲路、市北路二期、金戈路、体育路等城市支路，完成“一环四路”27公里综合管网入地、“一环两路”街景立面整治、5个人行过街设施和14公里道路“白改黑”工程，国庆前中心城区亮丽工程投入使用。城市化率提高1个百分点以上。金阳新区新的增长极作用进一步显现，建成观山公园、妇女儿童活动中心、青少年活动中心等一批公建项目，世纪金源大酒店建成开业。新区完成固定资产投资115.18亿元，增长36.7%；财政总收入12.21亿元，增长106%；销售商品房362.12万平方米，占全市总量的44.3%。

城市生态环境持续改善。成功举办2009生态文明贵阳会议。被列为全国生态文明建设试点城市。实现“两湖一库”水质持续恶化趋势得到遏止的阶段性目标。清镇、修文、息烽、开阳等一批县城污水处理厂建成，城市污水处理能力达62.6万吨/天，主城区污水处理率达100%。建成3个山体公园和2个绿化广场，完成营造林14.2万亩，新增绿地25.8万平方米，森林覆盖率比上年提高1个百分点以上。花溪湿地公园被列为国家城市湿地公园。城区空气质量良好以上天数稳定在95%以上。

（四）统筹城乡重“三农”，一体化发展有新局面

大力实施强农惠农政策，财政用于“三农”支出达21亿元。加强基础设施建设。整区（市、县）推进农村危房改造，67168户农村危房改造竣工率达90.6%；贵遵路沿线村庄整治基本完成；完成1100公里村寨串户路、500公里农村公路和20个乡镇客车站建设，改造基本农田3.2万亩、中低产田土3.99万亩，解决农村19.33万人饮水安全问题。切实推进农业产业基地化、标准化、规模化。基本完成2万头奶牛养殖基地建设，奶牛存栏数突破4万头，增长一倍。建成一批绿色、无公害优质蔬菜、水果、中药材、茶叶种植基地。肉、蛋、菜、奶产量超过上年，市场效益回升。贵阳国家农业科技园区通过科技部验收。扶贫开发取得实效。

（五）改革开放增活力，城市可持续发展动力有新增强

更大力度推进改革。深化投融资体制改革。搭建11个投融资平台公司，组建后的金阳公司、城投公司成功发行理财产品，预期筹资48亿元的企业债券发行工作进展顺利。深化行政管理体制改革。积极推进市级政府机构改革，探索建立职能统一的大部门体制。行政审批服务时限平均由承诺的22.6个工作日压缩到5.2个工作日以内。取消和停收239项行政事业性收费项目，减轻企业负担1.2亿元。深化国有企业改革改制。完成50户国企改革重组，企业政策性破产工作全部终结。集体林权制度改革加快推进。91%的集体林地明确了产权。

扎扎实实推进非公有制经济发展。非公有制经济完成增加值368.14亿元，增长16%，注册户数12.67万户，占全市注册总户数的94%。安置就业4.3万人。

努力扩大对内对外开放。推出107个投资总额达609.89亿元的城市基础设施招商项目，探索BT模式加快城市建设。组建六大重点产业招商工作组和港澳招商工作组。与全国16家海关开通“属地申报、口岸验放”通关模式，简化出口退税及外汇核销手续，缩短办理时间。积极开拓海外新兴市场成效明显，新兴市场整体出口3.3亿美元，增长43.6%。引进投资3000万元以上项目135个，亿元以上项目42个，引进内资实际到位资金368.52亿元,增长21.6%;实际直接利用外资1.12亿美元，增长20%。

（六）改善民生促和谐，各项社会事业有新进步

认真落实“六有”民生行动计划，十件实事全面完成。市本级统筹28.1亿元用于改善民生，增长15.1%，占财政总支出的52%。努力稳定和扩大就业。强化、细化大中专毕业生就业指导、小额贷款担保、就业援助、劳务协作等工作措施，37.7%的社区成为充分就业社区，“零就业家庭”动态为零。大力实施均衡教育。“两基”顺利通过“国检”，得到国家教育部高度肯定；在两城区探索义务教育阶段学区化管理；建成4所外来务工人员子女学校、8所农村标准化学校，对承担进城务工人员子女义务教育的民办学校给予生均公用经费补助。对贵阳户籍中职教育新生免除学费。市属高等教育强化内涵发展，教育质量进一步提高。完成市财校等四所中专整合升格工作。积极构建安全、有效、便民、价廉的公共卫生服务体系。建成5个标准示范社区卫生服务机构、6个示范乡镇卫生院，新建、改造一批乡镇卫生院和村卫生室。成功处置人感染高致病性禽流感事件，有效防控甲型H1N1流感和手足口病疫情。对城乡困难群众实现惠民医疗政策全覆盖。166.24万农民参加新型农村合作医疗，参合率达96%。稳步提高社会保障水平。城镇基本养老、基本医疗、失业、工伤、生育保险参保人数分别为77万人、151万人、38万人、51万人、72万人，新型农村养老保险参保人数达12.5万人，累计发放城乡低保资金1.56亿元，保障15.25万困难群众基本生活。加强保障性住房建设。开工建设廉租房28.99万平方米，竣工10.74万平方米；经适房在建192.5万平方米，竣工101.5万平方米；建成农民工公寓18.5万平方米。全力以赴保稳定。深入开展“严打两抢一盗”、“打黑除恶”等专项行动；实施“清积案、促和谐”两年化解信访疑难问题工作计划，479件积案已化解401件；完成构皮滩水电站开阳库区移民搬迁工作。严格落实安全生产责任制，狠抓安全隐患治理，连续8年获得全省安全生产工作第一名，市安监局被评为全国安全生产工作先进集体。低生育水平保持稳定。人口出生率为10.5‰，人口自然增长率为5.4‰，符合政策生育率为95.8%，出生人口性别比为107，保持在正常范围。连续15年获得全省人口和计生目标考核一等奖第一名，被评为全国人口和计划生育综合改革示范市。文化、体育事业取得新成绩。深化文化体制改革，完成经营性文化事业单位转制改企；提前6年在全省率先实现村村有农家书屋；新增1000个自然村广播站，新增电视入户37927户；推进社区信息化试点工作，建成100个农村信息超市；成功举办第七届中国舞蹈“荷花奖”民族民间舞大赛、亚洲青年动漫大赛并获得好成绩；组建贵阳交响乐团，是全国第一家民营资本投入的城市职业交响乐团；我市运动员在第11届全国运动会等重大赛事中获得好成绩。荣获全国民族团结先进集体。

（七）依法行政优服务，政府自身建设有新成效

加强依法行政，自觉接受人大法律监督、工作监督和政协民主监督，认真听取民主党派、工商联和无党派人士的意见，支持工、青、妇等人民团体开展工作。办结市人大代表建议160件，市政协提案354件，办结率100%，满意率98.6%。向市人大提交了3部地方性法规草案议案，出台9件政府规章。深入推进政务公开，坚持市政府新闻发布会制度，在全国率先推行市政府网络新闻发言人制度，就全市性重点工作和市民群众关心的政府工作广泛问政于民，最大程度保障人民群众知情权、参与权、表达权和监督权。强化审计监督和行政监察，加强市级机关公务用车、公务接待、公款出国（出境）等管理，公用经费支出大幅下降。武警支队新营房投入使用，对国防后备力量建设支持力度进一步加大，国防动员能力不断提高，军政军民团结得到巩固。老龄、宗教、人防、史志、档案、保密、侨务、对台、仲裁、气象等工作都继续有新进展。

2009年我市率先发展、科学发展的实践，生动展示了新中国成立60年来全市人民知行合一、协力争先的精神面貌，我市被评为建国60周年中国城市发展代表。事非经过不知难，在困难前所未有，挑战和冲击接踵而至的不利形势下，我市取得的成绩确实来之不易，这是全市人民在省委、省政府和市委的正确领导下，团结拼搏、奋力攻坚所取得的，值得倍加珍惜。在此，我代表市人民政府，向在各个领域和岗位上辛勤劳动、无私奉献的全体市民，向给予政府工作积极支持的人大代表和政协委员，向各民主党派、工商联、人民团体和各界人士，向中央及省在筑单位，向驻筑人民解放军、武警官兵和公安政法干警，向所有参与、支持和关心贵阳建设和发展的海内外朋友，表示崇高的敬意和由衷的感谢！

各位代表，今年经济发展环境总体好于去年，但不确定不稳定因素很多，促进我市经济社会又好又快发展仍然面临严峻挑战和困难：一是我市发展面临扩大经济总量和优化经济结构双重任务，资源型产业和低附加值产品比重偏大，产业结构不尽合理，受国内外形势影响，经济回升向好的基础仍不稳固。二是农民增收渠道不多，现代农业产业化程度不高，城乡发展差距仍在扩大，城乡一体化任务艰巨。三是资源要素和环境容量制约加剧，切实抓好节能减排、资源节约和环境保护需加大力度。四是民生方面需求多，尚有许多涉及群众切身利益的问题亟待解决，加之财政处于偿债高峰期，保障和改善民生压力大。这些问题，我们一定高度重视，认真加以解决。

2010年政府工作的目标任务

总体要求：贯彻落实党的十七届四中全会、中央经济工作会议和省委十届七次全会、全省经济工作会议、市委八届八次全会精神，高举中国特色社会主义伟大旗帜，以邓小平理论和“三个代表”重要思想为指导，深入贯彻落实科学发展观，积极抢抓国家新一轮西部大开发、我省加快推进西南交通枢纽建设的重大机遇，坚持好字优先、能快则快，着力推进经济结构调整，着力推动产业创新发展，着力提升城乡规划、建设和管理水平，着力深化改革扩大开放，着力改善民生，扎实抓好“三创一办”工作，切实增强执行力，全面完成“十一五”规划各项目标任务，纵深推进生态文明城市建设。

经济社会发展主要预期目标：全市生产总值增长13%，其中，第一产业增加值增长8%，第二产业增加值增长12%，第三产业增加值增长17%。财政总收入增长12%，地方财政收入增长12%。全社会固定资产投资增长30%。外贸进出口总额增长15%。社会消费品零售总额增长17%。城市居民人均可支配收入增长10%，农民人均纯收入增长10%。城镇登记失业率控制在4.5%以内。居民消费价格指数控制在3%以内。人口自然增长率控制在5.5‰以内。单位生产总值能耗比上年降低4%，主要污染物排放控制在省下达目标内。

为民办好的十件实事：

在金阳新区建成贵阳职业技术学院新校区（一期）和9所中小学及幼儿园并投入使用；云岩、南明各新建一所小学，主要用于接收进城务工人员随迁子女。

提高农民最低生活保障标准，其中：三县一市不低于1300元，花溪区、乌当区、白云区、金阳新区不低于1500元，云岩区、南明区、小河区不低于1800元；拓展社保覆盖面，确保养老保险扩面5万人、新型农保扩面5万人、城镇职工医保扩面4万人、城镇居民医保扩面3万人。

继续加大城镇廉租房保障和建设力度，确保开工建设24万平方米，竣工5万平方米，完工1000套，将人均住房建筑面积不足15平方米的城镇低收入群体纳入廉租住房保障范围；开工建设公共租赁房1.2万平方米，200套；在金阳新区建成50万平方米拆迁安置房。

完成村寨串户路建设项目1000公里、村级公路800公里和80个规模养殖企业大中型沼气池工程建设及排污设施建设，完成农村清洁工程20个，逐步解决养殖企业面源污染问题。

开工建设北二环、东二环、北京东路、甲秀中路、甲秀北路、开阳至息烽高速公路、白修线、金阳至清镇城市干道和金阳客运站、将军山货运站和贵阳客运南站，改造210国道三桥至粑粑坳路段；新增停车位3000个。

继续实施电力管网入地工程，花溪大道景观环境综合整治工程；云岩、南明各翻修背街小巷破损道路25条；新增人行过街系统3处；新建和改扩建公厕60座、垃圾台40座、清洁间800个、环卫停车场2.8万平方米、配置果皮箱3500个；建设10个城郊结合部社区便民利民综合服务站。

继续加大两湖一库污染源治理力度，启动红枫湖污染底泥环保疏浚；启动花溪国家级十里河滩生态湿地公园建设；改造市西河流域、贯城河流域雨污分流系统；基本建成10万吨北郊水厂、南郊垃圾填埋场；开工建设二桥污水处理厂二期、花溪南部污水处理厂和自来水厂；营造林15万亩，金阳新增绿地10万平方米；异地搬迁贵州水泥厂。

政府开办的社区卫生服务机构及乡镇卫生院、村卫生室实行国家基本药物零差率销售；建立居民健康档案信息系统。

完善报警与监控管理系统，增加城市报警管理监控点5000个，开工建设公安技术综合大楼；为困难群众无偿办理法律援助案件2500件以上，提供义务法律咨询10000人次以上。

建成奥体中心主体育场、市民健身中心、1个文化馆和22个综合文化站；新建10公里公园登山步道和2个山体公园；继续实施农村广播电视户户通工程，新增28350户农户通电视。

围绕以上要求和目标，今年要重点推进八个方面工作：

（一）着力增加投资扩大消费，保持经济平稳较快增长

促进固定资产投资强劲增长。充分发挥投资对经济增长的拉动作用，实施政府主导类重点建设项目76个，促进民间投资快速增长，确保全社会固定资产投资总额突破1000亿元，完成政府投资项目“投转固”45项。立足提升我市在西南城市体系中地位加快基础设施建设，完成投资240亿元。抢抓省委、省政府加快建设贵阳通往全国七小时快铁交通圈机遇，积极配合省抓好贵阳至广州、重庆、昆明、长沙、成都等快速铁路和沪昆高速公路，龙洞堡国际机场二期扩建，开阳港区、息烽港区等对外交通体系建设。加快推进市域快速铁路网工程，城市轻轨一号线全线动工，会展中心车站主体工程年内完工，完成铁路、轻轨及配套设施投资60亿元。开工建设“两路二环”、盐沙线及开发大道三期，加快建设清镇至织金高速公路，开工建设清镇城区至清镇煤化工、铝加工循环经济生态工业园高等级公路，做好清镇至黔西高速公路前期工作。强化电网基础设施建设，确保一批关键变电站及线路建成投入运行，完成电网建设投资25亿元。立足产业调整振兴加快重大产业项目建设，完成投资300亿元。扎实推进首钢贵阳特殊钢新特材料循环经济工业基地、贵航军转民高新技术产业园、华电清镇塘寨电厂2×60万千瓦机组、贵州广铝年产80万吨氧化铝等一批续建重大工业项目建设。抓好39个亿元以上产业项目建设，完成工业投资160亿元。滚动完善产业项目库，分产业制定进库项目投资规模、产出强度、环境效益、财政贡献、就业机会、带动效应等标准，确保产业项目库投资规模保持500亿元以上，高新技术产业和新兴产业投资规模占项目库总量的30%以上。立足“三创一办”抓好公建项目建设及环境整治，完成投资34亿元。做好协办全国民运会的相关体育场馆建设、改造工作。兴建省危险废弃物暨市医疗废弃物处理处置中心，实施新庄污水处理厂中水回用工程，实施清镇污水处理厂二期工程。继续加强“两湖一库”等重要饮用水源地保护与治理，抓好南明河全流域综合治理。

努力扩大消费需求。积极促进外来消费。以避暑季、温泉月为载体，办好药博会、农博会、亚洲青年动漫大赛、花溪之夏艺术节、美食节、购物节等系列活动，加快乌当生态体育公园、清镇红枫湖国际休闲体育运动公园、修文苏格兰牧场二期等一批高档旅游项目建设，建成贵阳国际会展中心、凯宾斯基酒店、希尔顿酒店、保利国际温泉度假酒店、乌当国际温泉城，吸引更多游客到贵阳消费。稳定扩大住房消费。启动10个城中村改造项目。实施一批棚户区改造试点，力争三年完成全市棚户区改造。全面完成农村危房改造任务。增加中低价位、中小套型普通商品房供应，对符合条件的低收入群体购房实行货币直补，创造条件让大学生就业十年就有能力购买商品房。修订物业管理地方性法规。联合房开企业到外地开展“避暑之都购房休闲游”活动，推动旅游地产开发。繁荣农村消费市场。继续落实家电下乡、汽车摩托车下乡、农机具购置补贴等政策，支持商贸流通企业向农村延伸经营网络，提高农资超市和农家店覆盖面，促进农村消费。优化市场环境和消费环境。强化市场价格监测，加强涉及民生的价费监管，加大产品质量和食品药品安全专项整治力度，营造安全、放心的消费环境。

（二）深化产业结构调整，不断形成新的发展优势

建设创新型城市。加快重点产业、企业创新步伐。实施重大科技专项15—20个，重点科技项目50个，产业技术创新项目40个。支持企业新建工程技术中心15家，新建产业技术创新联盟5个，建设科技基础条件平台2—3个。力争国家级创新企业孵化器挂牌，建成中国·西部（贵阳）高新技术产业研发生产基地一期。实施人才强市战略。建立统一规范、满足不同需求的人力资源市场，大力引进高层次人才、创新团队和产业发展急需高技术人才，在高新区建设留学归国人才创业园，营造有利于人才集聚和创业创新的良好环境。实施知识产权、标准和品牌发展战略。编制完成自主知识产权产品目录，进一步加强专利申请、保护工作，打造酒类及食品加工、无机磷化工产品、建筑材料产品及机械/电子基础原器件产品等四个国家级质量检测中心。

促进服务业提质增量。推动旅游业上台阶、上水平。召开第四届旅游发展大会。组织赴重庆、广州、武汉、南昌等城市开展旅游推介，加大青岩古镇、乌当温泉度假旅游中心、开阳“十里画廊”旅游区、修文阳明风景名胜区、息烽集中营等旅游精品区开发力度，抓好天邑森林温泉4A级、天河潭景区5A级旅游区创建工作，培育壮大乡村旅游集群。旅游人数增长20%，旅游收入增长40%。加快区域性金融中心建设。继续实施“引银入筑”工程，促进花旗银行、浦发银行等6家银行贵阳分行挂牌开业。成立贵阳市农村商业银行，设立一批村镇银行，强化农村金融服务。做好市商业银行上市和跨区域经营相关工作。畅通银政企沟通渠道，鼓励金融机构加大对骨干企业、重点项目和企业重组的支持，努力化解中小企业融资难题。严厉打击非法金融活动。加快发展现代物流业。创建全国流通领域物流示范城市，规划建设小河“无水港”，搭建物流信息资源共享平台，启动二戈寨物流配送基地、金阳物流园区、清镇物流园区等项目建设。加快发展商贸流通业。大力培育电子商务企业，规范发展网络销售等新兴销售业。建成世纪金源大型购物中心和会展中心城市综合体。继续实施“万村千乡市场工程”，加快推进“双百”市场和标准化菜市场建设，升级、改造一批农贸市场。大力发展新兴服务业。培育总部经济、楼宇经济，提升法律咨询、会计审计、工程咨询、信用评估等服务业发展水平。加强规划引导，重点发展动漫游戏、现代传媒、软件设计，动漫软件等创意产业增长50%。

坚持大调整大开放大实干振兴工业经济。培育、壮大优势产业。磷化工、铝加工、煤化工方面，重点抓好安达公司30万吨有机磷配套氯碱、开阳化工公司100万吨醇氨、开磷集团30万吨合成氨二期、中铝贵州分公司高性能铝合金复合材料、贵州今飞公司铝轮毂、华能焦化公司五号焦炉等重点项目建设，促进开磷集团120万吨磷酸二铵工程二期、华能焦化四号焦炉技改等一批重点项目达产增效。努力提高资源就地转化和利用率，确保重点工业企业污染物排放稳定达标。装备制造业方面，重点抓好詹阳公司特种工程机械国产化、贵州轮胎公司年产110万条全钢子午线轮胎技改、航天林泉电机公司节能型复式永磁电机生产线异地技改、永青公司年产10万台工程机械电子监控系统生产线技改等重点项目建设。烟草及特色食品、现代药业方面，促进贵阳卷烟厂异地技改、老干妈春梅酿造公司二期技改等重点项目达产增效，突出抓好益佰制药工业园、贵阳酒厂万吨技改、益康集团GMP生产基地及营销管理中心等项目建设，组建贵州苗药集团，制药业产值增长20%。战略性新兴产业方面，重点抓好贵阳汇通膜科技公司复合反渗透膜及装备国产化、振华新材料5000吨锂离子正极材料、振华云科片式薄膜电阻器生产线技改、宝源阳光公司年产800吨太阳能极多晶硅产业化等项目建设，大力支持朗玛科技公司开拓网络通信、游戏市场，促进益佰、信邦等生物制药项目做大做强，市工业技改资金和市应用技术研发资金重点支持绿色环保和新兴产业的开发和建设，力争在新材料、新能源、电子信息、生物医药等领域取得突破性进展。确保工业增加值增长13%，高新技术产业增长20%。加快重点企业发展。按照“重点扶持、动态更新”原则，继续抓好四个“十大”重点企业和重点工业项目，加快建立现代企业制度。培育1家年产值超百亿元的大型企业集团，发展一批10亿至50亿元的骨干企业，确保2家企业在主板上市。加快工业园区建设。统筹推进园区征地、场平和水、电、路、气、通信等基础设施建设。积极采取BT、银行融资等方式，筹资用于园区基础设施建设。重点抓好麦架—沙文高新技术产业园、小河—孟关装备制造业生态工业园、清镇煤化工铝加工循环经济生态工业园、开阳磷煤化工生态工业示范基地、息烽精细磷煤化工基地、龙洞堡特色食品工业园建设。建成5万平方米标准化厂房。实施工业园区产业规划，新引进项目原则上一律入园发展。

（三）以“三创一办”为重点，努力建设宜居宜业宜游城市

做深做细城乡规划。围绕《贵阳市城市总体规划（2009—2020年）》、《贵阳市土地利用总体规划（2006—2020年）》，抓紧完善环城高速公路内各城市功能片区控制性详细规划和细部设计，高水平做好客货运场站、大型专业市场、中心城区环卫设施等专项规划，增强各类规划的协调性。

强力实施城市拓展计划。以环城高速公路及“五路”通车为契机，开工建设金竹、盐沙、水东等三个立交连接工程，以路网建设为龙头，重点抓好各片区配套设施建设和功能完善，加快城市拓展步伐。新增城市面积20平方公里，城市化率提高1个百分点以上。大力提升中心城区品质。重点实施遵义路等18条主、次干道街景整治，公园路、文昌路“白改黑”等工程。加强城市夜景规划和建设，实施市区各主干道、甲秀楼—南明河等重点景观区域的二期亮丽工程，加强路灯等市政设施的管理维护，确保城市景观灯亮灯率达95%，路灯亮灯率达98%。金阳新区重点抓好茅台大厦、烟草大厦、盘江煤电大厦、开磷集团大厦、贵州移动大厦等一批总部大楼建设，开工建设贵阳北站前广场等一批重大配套设施，加快金阳十二滩地块整体开发，努力形成更便捷的公交体系，一批大型超市、步行街开业，努力促进新区产业发展和人气、商气聚集。做好在建道路周边土地储备和城市设计，加快新开通道路周边地块开发，建设太慈桥片区路网，加快中心城区大运载量的市场、企业搬迁，建设孟关汽车商贸城等市场，促进东部新城、三马片区与中心城区、金阳新区融合发展。

提高城市精细化管理水平。全面推行数字化、网格化城市管理模式，积极发挥数字化城市综合管理系统作用，增强城市管理服务的快速反应、快速处理能力。综合疏“堵”。逐步建立集交通监控、信号控制、及时诱导等功能于一体的交通管理智能系统，规范停车场点建设，严格交通执法，提高市民驾车良好行为率；继续实施公交优先战略，加大公交场站、专用道建设。实现城区出租车运营一体化，适度增加出租车数量，加大“禁摩”和打击非法营运力度，全面提高公交覆盖率和满意率。强力整“脏”。城区运渣车、垃圾车确保外观清洁并封闭运输、限时上路，提高道路机械清扫率，严格“门前三包”，加强城郊结合部、城中村、铁路沿线、背街小巷市容环境整治。突出治“乱”。综合治理占道经营、乱搭乱建和城市“牛皮癣”、“都市陷阱”等城市顽症，形成依法治理长效机制。创新城市管理体制。推行政府购买市政服务改革试点，将城区部分主干道的市政绿化、保洁等工作通过招投标交由专业公司管理。探索城市管理新模式，整合行政执法资源，实现管理重心向街道、社区下沉。深入开展文明乡镇、社区、学校、医院等创建活动，开展绿丝带志愿活动，提高城市文明程度。深入查找城市建设、管理中存在的问题和不足，以攻坚精神狠抓整改，落实责任，确保在规定时间内指标全部达标，力争获得国家卫生城市和国家环境保护模范城市称号，为创建全国文明城市和协办好全国民运会奠定基础。

（四）促“四化”强“三建”，推进城乡一体化发展

促进农业产业规模化。加大对龙头企业的扶持和培育力度，重点扶持50家市级龙头企业，培育10家产值超亿元的龙头企业。进一步完善农村土地承包经营流转方式，帮助农民实现就地就业，探索耕地得到切实保护、发展用地得到切实保障、土地效益得到切实发挥、农民权益得到切实维护的土地流转新格局。促进农业产业特色化。继续大力实施畜、禽、蛋、奶、果、蔬、花、药、茶等现代农业示范工程，加强国家级农业科技示范园区建设，重点打造花溪特色农产品加工市场。新建4个大型奶牛养殖场，建设一批中药材基地、花卉基地、无公害果树基地、无性系茶园和规模生态蔬菜基地。促进农业生产标准化。加强无公害农产品、绿色食品和有机食品示范基地建设和认证，完善10个县级农产品检测中心，确保主要农产品基地产品抽检合格率达98%以上。促进城乡公共服务均等化。着力完善农村教育、卫生、文化、体育等公共服务体系，健全农村垃圾处置体系，改善村级档案室设施条件。坚持开发式扶贫、开放式扶贫和救助式扶贫，对有劳动能力的农村低保对象全面实施扶贫。

加强农业基础设施建设。重点抓好鱼洞峡水库建设，开工建设席关水库、金龙水库等一批骨干水利工程，实施乌当中型灌区等节水改造，完成一批病险水库除险加固和重点水源区水土保持治理工程。重点解决3.53万农村人饮安全问题。加强城镇化建设。注重城镇规划、载体建设、产业调整的对接、融合，提高修文、开阳、息烽县城和清镇市区的综合承载能力，加快站街、扎佐、双流、小寨坝等中心镇规划建设，提高产业聚集和人口吸纳能力。深化户籍制度改革，推行居住证制度，促进农民向城镇集中。加强新农村建设。巩固提高12个省级示范点、38个市级试点和10个精品民族村寨的创建成果，全力抓好100个重点村庄规划、建设。

(五)大力发展绿色经济、循环经济、低碳经济，进一步强化生态优势

加强林业绿化建设。重点搞好环城林带等森林资源、野生动植物及绿地资源的建设、保护和利用，抓好贵遵、贵新、贵毕高等级公路境内沿线景观绿化建设，新增绿地面积45万平方米以上，确保森林覆盖率提高1个百分点以上。先行先试开展排放权交易。支持阳光产权交易所有限公司创建贵州环境交易所，率先开展环境权益交易试点，打造成国内知名的排放权交易平台。力争开阳户用沼气打捆碳贸易项目在国外拍卖成功，让人民群众享受到碳减排实惠。发展循环经济、低碳经济。完成贵州轮胎公司蒸汽供热系统优化节能改造、黔能天和黄磷尾气发电等项目建设，继续实施开阳紫江公司磷渣水泥、新强公司回收利用黄磷尾气年产5000吨甲酰胺、水晶公司电石渣综合利用等项目，加快推进贵阳钢厂、清镇发电厂等重点污染源企业异地改造，淘汰一批不符合产业政策和环保要求的落后产能。积极发展绿色建筑，严格执行建筑节能标准，推广使用节能环保建筑材料。倡导绿色生活方式。加强宣传教育，重点办好生态文明贵阳会议，开展低碳社区、低碳学校试点，推广使用节能灯等清洁能源产品，倡导每周少开一天车，使绿色生活方式、消费模式深入人心。

（六）深入推进改革开放，着力创新体制机制

推进重点领域改革。深化行政管理体制改革。上半年完成核定市政府各部门“三定”工作。加强各级政务服务中心建设，深入推进行政审批制度改革和电子政务，提高行政效能。深化财政体制改革。积极开展综合治税工作，调整完善市、区（县、市）两级政府收入分配关系。深化投融资体制改革。积极拓宽融资渠道，采取多种融资方式筹集建设资金。力争金阳公司、城投集团企业债券成功发行。完善风险预警与偿债机制，促进11家投融资平台公司加快形成“融投建、借用还”一体化机制，实现融资100亿元。基本完成国企改革。完成30户国企改革任务，将国资管理重心向加强监管和实现国有资产保值增值转变。完成市自来水总公司资产重组工作，力争组建水务集团，实现上下水运营一体化和城市自来水管网一体化。稳步推进城镇供水、污水处理、垃圾处置、管道煤气等公用事业价格改革。

扶持非公经济和中小企业发展。认真落实支持非公经济和中小企业发展的政策措施，强化社会化服务体系建设，重点加强融资担保、技术创新、检测检验、人才培训、信息咨询等服务。鼓励、帮助中小企业与大企业“联姻”，中小企业向“专、精、特、新”方向发展，围绕大企业上下游产业链提供协作配套。重点培育20家市场潜力大的中小企业，力争3家企业在创业板上市，打造一批年产值过亿元的科技小巨人企业。

提高对内对外开放水平。加强与长三角、珠三角、京津唐、成渝等地区的经贸交流，赴东南亚、东亚、台湾及北京、上海等地开展经贸推介，承接发达地区产业转移。力争引进1至2家世界500强和3至4家国内500强企业落户贵阳。支持机电产品、高新技术产品及特色食品出口，积极拓展中东、非洲、东欧、中亚、东盟等新兴国际市场，申报建设贵阳综合保税区，促进外贸进出口平稳增长。

（七）切实改善民生，推进社会全面进步

千方百计抓好就业和社会保障工作。深入创建国家级创业型城市，以创业带动就业。就业方面，确保新增城镇就业4.5万人，转移农村富余劳动力2.5万人，开发公益性岗位4500个，城镇登记失业率控制在4.5%以内，40%的社区建成“充分就业社区”，继续保持“零就业”家庭动态为零。社会保障方面，提高城乡低保标准，强化社保基金监管。实现失业保险扩面3万人、工伤保险扩面3.5万人、生育保险扩面4万人。

加快教育均衡发展。强化薄弱学校建设，提高教育教学质量和水平，落实中小学校长、教师交流制度和学区内划片入学制度，推动义务教育均衡发展，逐步消除“择校”现象。新建6所农村寄宿制标准化学校，加强省示范性高中建设，实施“校安工程”。加快发展职业教育，对贵阳市户籍学生就读本市中职学校（含技工学校）继续免除学费，支持贵州工商职业学院、省旅游学校异地搬迁和省电力职业学院生产技能实训基地建设，抓好职教中心建设。坚持高等教育内涵式发展方向，支持花溪高校聚集区和贵州商专白云校区建设，提升贵阳学院办学水平。

大力发展医疗卫生事业。扎实推进医药卫生体制改革，逐步将社区卫生服务机构建设成为财政全额拨款的公益性事业单位。组建市公共卫生救治中心，进一步完善重大传染性疾病防控体系。加快市妇幼保健院综合病房大楼、市一医医技综合楼、市口腔医院新大楼、金阳医院新综合楼、疗养康复基地等项目建设，建成3个县级人民医院扩建主体工程，新建4个社区卫生服务中心并投入使用。启动市域卫生信息网络建设。围绕控量提质，继续稳定低生育水平，抓好流动人口计生管理服务、“奖、扶、医、帮、优、保”利益导向、综合治理出生人口性别比偏高、优质服务等八大亮点工作，保持人口和计划生育工作领先水平。

切实维护社会稳定。深入开展“严打两抢一盗”、“打黑除恶”、禁毒等专项行动，健全完善扁平化指挥网格式警区防控机制，加强社会治安综合治理。做好信访工作，力争479件信访积案化解完毕，建立健全人民调解、行政调解、司法调解三位一体的“大调解”工作体系，及时疏导和化解矛盾纠纷。进一步抓好移民后期扶持政策的落实，解决好构皮滩水库开阳库区、洪家渡水电站迁入移民及失地农民长远生计问题。进一步完善应急管理体制机制，加强基层应急管理工作基础，不断提高预防和处置突发事件的能力。严格落实安全生产责任制，严查安全事故隐患，确保安全生产事故发生起数、死亡人数控制在省下达目标之内。

加快文化体育等社会事业发展。进一步深化文化和广播电视体制改革，推进文化进社区（村）、非物质文化遗产保护等工作，加强“两馆一站”建设。推进“两台一站”数字化。抓好全民健身工作，办好市第11届运动会，精心挑选并强化少数民族运动项目运动员培养工作。进一步加强气象防灾减灾工作，提高气象灾害的防御能力。

（八）以增强行政执行力为核心，加强政府自身建设

转变政府职能。更好地履行经济调节和市场监管的职能，积极应对国际金融危机，加强对苗头性、倾向性问题的研究，做好经济预测预警工作。特别要立足抓“早”落实好资金、土地，财政性资金要重点保续建工程、保中央扩大内需项目、保“三创一办”急需项目；加快推动银政企对接，新开工项目争取在第一季度落实好资金盘子；对急需建设用地抓紧报批，已落实的项目优先安排土地。进一步加强社会管理和公共服务职能，始终坚持“立党为公、执政为民”，按照“群众生活中的小事，就是政府工作的大事；群众生活中的难点，就是政府工作的重点”的要求，做到“民有所呼、我有所应”，努力为群众办好事、办实事、解难题，扩大政府公共服务的覆盖范围，提高公共服务的供给能力和质量，确保服务到位。紧扣我市发展实际，以生态文明城市建设为主线，编制好“十二五”规划。

加强依法行政。全面落实“五五”普法依法治市规划，深入推进依法治市工作。自觉接受市人大及其常委会的法律监督和工作监督，自觉接受市政协的民主监督，认真听取民主党派、工商联、无党派人士和各人民团体的意见。认真做好人大代表建议、政协提案的办理工作。坚持政府信息“公开是常态，不公开是特例”，深入推进政务公开，重视新闻舆论和社会公众监督，全面推行重大决策事项公示、听证制度，健全专家咨询制度，推进决策科学化、民主化、制度化。

全面加强廉政建设。认真落实党风廉政建设责任制，扎实推进惩治和预防腐败体系建设，健全建筑工程、土地交易、产权交易、政府采购等重点领域监管的长效机制。强化行政监察、财政监管和审计监督。强化对涉农、教育、卫生、社保基金等方面资金的监督管理，保障各项惠民政策落实到位。厉行勤俭节约，继续实现公用经费、行政购车经费、出国考察经费等支出“零增长”。

各位代表，今年是实施西部大开发战略10周年，是我市全面完成“十一五”规划的最后一年，困难和挑战考验着我们，责任和使命激励着我们，全面做好今年工作、促进经济社会又好又快发展意义重大、任务艰巨。让我们更加自觉地按照胡锦涛总书记“做表率、走前列”的要求，抢抓机遇，开拓奋进，纵深推进生态文明城市建设，为早日将贵阳建设成为走新型工业化、城镇化道路的先行区，深化改革、扩大开放的试验区，建设生态文明、精神文明的示范区而努力奋斗！
